# DET1 and HY5 Control PIF4-Mediated Thermosensory Elongation Growth through Distinct Mechanisms

**DOI:** 10.1016/j.celrep.2016.12.046

**Published:** 2017-01-10

**Authors:** Sreeramaiah N. Gangappa, S. Vinod Kumar

**Affiliations:** 1 Cell and Developmental Biology Department, John Innes Centre, Norwich NR4 7UH, UK

**Keywords:** thermosensory growth, environmental signal integration, elongation growth, *Arabidopsis*, *PIF4*, *DET1*, *COP1*, *HY5*

## Abstract

Plant growth and development are defined by environmental cues. The transcription factor PHYTOCHROME INTERACTING FACTOR 4 (PIF4) is the central signaling hub that integrates environmental cues, including light and temperature, to regulate growth and development. The thermosensory mechanisms controlling the PIF4-mediated temperature response, and its integration with other environmental responses, remain poorly understood. DE-ETIOLATED 1 (DET1) and CONSTITUTIVE PHOTOMORPHOGENESIS 1 (COP1), key regulators of light signaling, have been proposed to control thermosensory growth by transcriptional regulation of PIF4, through ELONGATED HYPOCOTYL 5 (HY5). Here, we show that DET1/COP1 and HY5 regulate thermosensory elongation through distinct mechanisms. DET1 and COP1 are essential for promoting PIF4 expression and stabilizing PIF4 protein. Furthermore, HY5 inhibits elongation growth through competitive chromatin binding to PIF4 targets, not through transcriptional regulation of PIF4. Our findings reveal a mechanistic framework in which DET1/COP1 and HY5 regulatory modules act independently to regulate growth through the environmental signal integrator PIF4.

## Highlights

• DET1 and HY5 control temperature-induced hypocotyl growth through distinct mechanisms
• HY5 is dispensable for DET1/COP1-mediated elongation growth at elevated temperatures
• DET1 and COP1 are essential for PIF4 protein accumulation
• HY5 regulates PIF4 target genes through chromatin binding at lower temperatures

## Graphical Abstract

**Figure:**
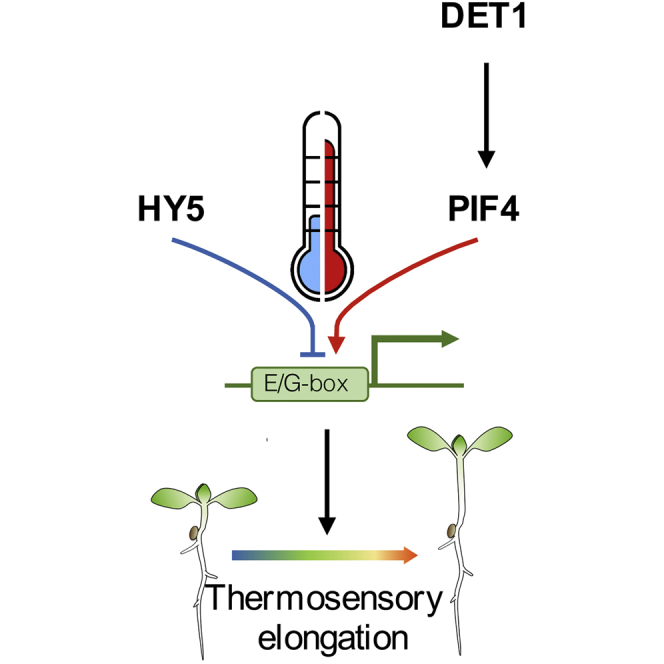

## Teaser

Gangappa and Kumar show that integration of light and temperature signaling is critical for growth. They show that major light signaling components DET1, COP1, and HY5 control PIF4-mediated thermosensory growth through distinct mechanisms. DET1/COP1 promote temperature-responsive growth by stabilizing PIF4 protein. HY5 antagonizes PIF4-mediated elongation through competitive chromatin binding.

## Introduction

The ability of plants to sense and integrate diurnal and seasonal changes in environmental signals, such as light and temperature, and accordingly modulate growth and development is critical for adaptation. Temperature and light being the most dynamic parameters play a pivotal role in plant growth and development. While the molecular mechanisms controlling response to light are well established (Jiao et al., 2007, Xu et al., 2015), those required for perception and response to temperature remain poorly understood (Quint et al., 2016). Phytochrome interacting bHLH transcription factors (PIFs), particularly PIF4, has emerged as a central signaling hub controlling thermosensory growth and development as well as coordinating environmental responses (Koini et al., 2009, Kumar et al., 2012, Leivar and Monte, 2014, Leivar and Quail, 2011). Despite its key role in thermosensory responses, the molecular mechanisms that control growth regulation by PIF4 and facilitate environmental signal integration remains to be fully elucidated.

Seedling hypocotyl elongation is a key trait of great adaptive significance, which is strongly influenced by environmental signals such as light and temperature (Quint et al., 2016). PIF transcription factors, particularly PIF4, coordinate elongation growth in response to environmental cues (Leivar and Monte, 2014). PIF-mediated growth is defined by a coincidence mechanism, wherein circadian clock regulates gene expression and light regulates protein abundance, which underlies photoperiod-dependent diurnal growth pattern (Niwa et al., 2009, Nozue et al., 2007). Photoreceptor Phytochrome B (PhyB) controls light-dependent regulation of PIF protein levels (Leivar and Monte, 2014, Leivar and Quail, 2011). Recent studies have further highlighted the tight coordination of light signaling and temperature responses. Key light signaling components that define photomorphogensis such as DET1 and COP1 have been proposed to control thermosensory growth by transcriptional regulation of *PIF4*, through ELONGATED HYPOCOTYL 5 (HY5) (Delker et al., 2014). This has further provided a regulatory framework for integrating light and temperature signaling. Despite these advances, the precise molecular mechanisms that control PIF4-mediated thermosensory responses is not clearly known. In this study, we undertook experiments to further dissect the molecular mechanisms by which DET1, COP1, and HY5 contribute to the thermosensory responses. Here, we show that DET1 and COP1 promote temperature-responsive elongation growth through promoting *PIF4* expression and stabilizing the protein. Further, our data demonstrate that HY5 negatively regulates PIF4-mediated elongation growth through competitive binding to the PIF4 targets gene promoters, not through transcriptional regulation of *PIF4*. Together, our study provides a mechanistic framework for the regulation of PIF4-medated thermosensory elongation growth.

## Results and Discussion

### Thermosensory Hypocotyl Growth Is Photoperiod Dependent

**Figure 1:**
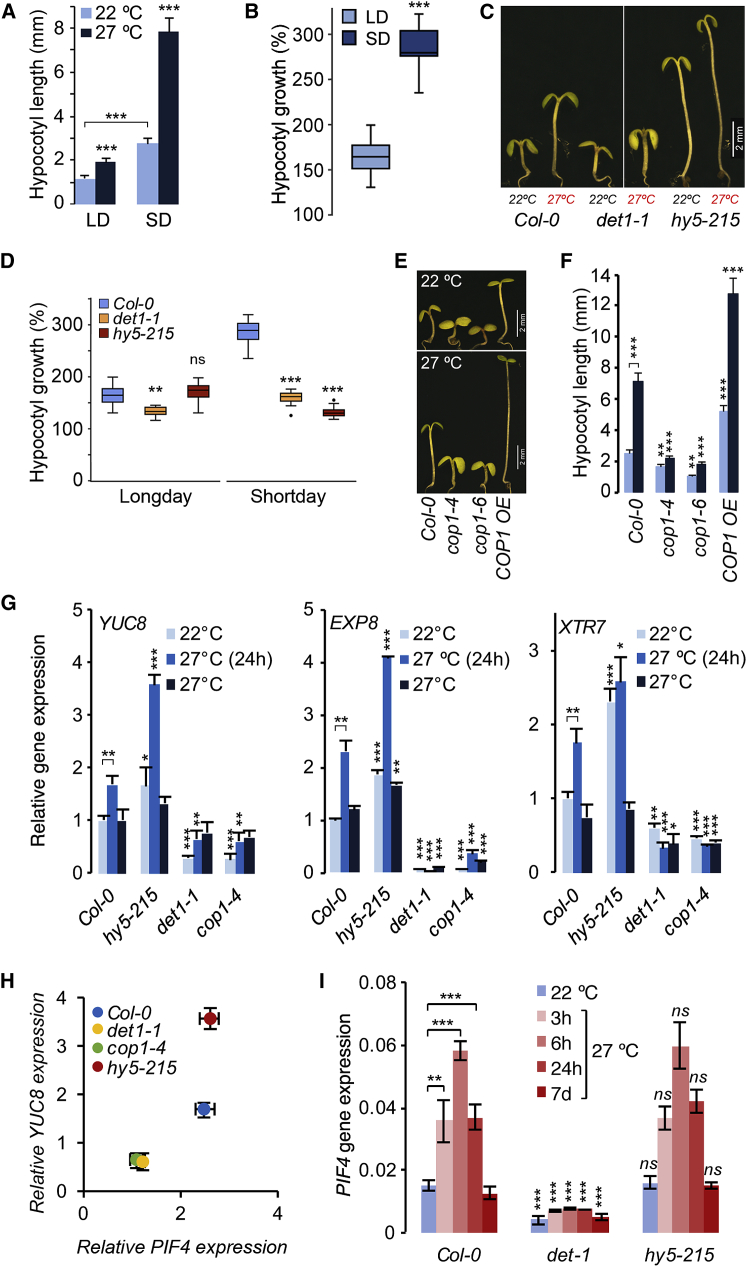
DET1 and HY5 Control Elongation Growth through Distinct Mechanisms (A and B) Thermosensory hypocotyl elongation growth is photoperiod dependent. Hypocotyl length (A) and percentage (%) hypocotyl response (B) of Col-0 seedlings grown in long-day (LD) and short-day (SD) photoperiod at 22°C and 27°C shows strong influence of day length on hypocotyl elongation and temperature response. Seven-day-old seedlings were used to measure hypocotyl length (mean ± SD; n ≥ 20). ^∗∗∗^p ≤ 0.001 (Student’s t test) significantly different from Col-0 in corresponding photoperiod conditions or between indicated pair. See also Figure S1. (C) Thermosensory hypocotyl elongation response is controlled by DET1 and HY5. Representative image of seedling hypocotyl elongation in 7-day-old *det1-1* and *hy5-215* along with wild-type (Col-0) grown at constant 22°C and 27°C under short-day photoperiod. See also Figures S1 and S2. (D) Hypocotyl elongation response (percentage growth at 27°C as compared to 22°C) of Col-0, *det1-1*, and *hy5-215* at 27°C (mean ± SD; n ≥ 20). ^∗∗^p ≤ 0.01, ^∗∗∗^p ≤ 0.001 (Student’s t test) significantly different from Col-0 in corresponding photoperiod. ns, not significantly different from Col-0. See also Figures S1 and S2. (E and F) Hypocotyl elongation is compromised in *cop1-4* mutant, whereas *COP1-OE* (*35S:COP1*) display enhanced thermosensory elongation showing the positive regulatory effect of COP1 on hypocotyl growth. Representative seedling picture (E) and the hypocotyl measurement data (F) of 7-day-old Col-0, *cop1-4*, *cop1-6*, and *COP1-OE* (*35S:COP1*) seedlings grown at constant 22°C and 27°C under short-day photoperiod are shown. ^∗∗^p ≤ 0.01, ^∗∗∗^p ≤ 0.001 (Student’s t test) significantly different from Col-0 in in corresponding temperature conditions or between indicated pairs. See also Figure S3. (G) Expression of growth-related genes *YUC8*, *EXP8*, and *XTR7* in Col-0, *det1-1*, *cop1-4*, and *hy5-215* mutants as measured by qRT-PCR (mean ± SD of three biological replicates) in 7-day-old seedlings grown constantly at 22°C, after 24-hr incubation at 27°C, as well as continuous growth at 27°C in short-day conditions. ^∗^p ≤ 0.05, ^∗∗^p ≤ 0.01, ^∗∗∗^p ≤ 0.001 (Student’s t test) significantly different from Col-0 in corresponding temperature conditions or between indicated pairs. See also Figure S3. (H) Expression correlation of *PIF4* and *YUC8* in Col-0, *det1-1*, *cop1-4*, and *hy5-215* genotypes. (I) *PIF4* is strongly downregulated and its temperature-responsive expression is attenuated in *det1-1*, while *hy5-215* shows wild-type expression levels (mean ± SD of three biological replicates). Seedlings either constantly grown at 22°C or transferred to 27°C for indicated time period or grown at constant 27°C in short-day conditions were used to measure the transcript. ^∗∗^p ≤ 0.01, ^∗∗∗^p ≤ 0.001 (Student’s t test) significantly different from Col-0 in corresponding time points or indicated pairs. ns, not significantly different from Col-0. See also Figure S3.

To understand the mechanism of integration of light and temperature signals, we studied seedling hypocotyl elongation in *Arabidopsis*. PIF4-mediated elongation growth is defined by a coincidence mechanism, which underlies photoperiod-dependent diurnal growth pattern (Niwa et al., 2009, Nozue et al., 2007). To test whether thermosensory growth is influenced by the same regulatory principles, we studied the influence of day length on temperature-induced hypocotyl elongation (Figures 1A and 1B). Col-0 seedlings grown in short day show pronounced hypocotyl growth and robust temperature-responsive elongation compared to long day, where hypocotyls were shorter and showed only marginal temperature response (Figures 1A and 1B). Moreover, we found that the photoperiod dependent effects on thermosensory growth is a general phenomenon across different genetic backgrounds (Figure S1), illustrating the strong coordination of growth by light and temperature signaling. These results clearly show that extended night period is conducive for temperature-responsive elongation growth. PIF4 constitutes the central regulatory module that define growth in response to light and temperature, and the underlying molecular mechanisms by which temperature is perceived and integrated with other signaling pathways such as that of light are not well understood. Therefore, to understand the regulatory framework that control PIF4-mediated growth, we sought to investigate the influence of light signaling components in thermosensory responses.

### DET1 and HY5 Control Temperature-Responsive Elongation Growth Likely through Distinct Mechanisms

The key photomorphogenesis regulator DET1 (Lau and Deng, 2012, Pepper et al., 1994) has been shown to promote temperature-induced hypocotyl growth by transcriptionally regulating *PIF4* at elevated temperature, through inhibiting HY5 in long days (Delker et al., 2014). We sought to examine whether the same regulatory hierarchy defines the photoperiod-dependent thermosensory elongation. Consistent with the earlier finding, under short days, *det1-1* showed characteristically short hypocotyl, and severely attenuated temperature-responsive elongation (Figures 1C, 1D, and S1B), confirming that DET1 is essential for thermosensory growth as reported (Delker et al., 2014). Interestingly, with its characteristically long hypocotyl, *hy5-215* showed only a modest increase (∼50% as opposed to ∼200% increase in Col-0) at 27°C (Figures 1C, 1D, and S1B). This was in contrast to the recent study, where *hy5* mutant showed only marginally elongated hypocotyls at normal temperature and an exaggerated response at elevated temperature in long days (Delker et al., 2014). To address this further, we analyzed four different *hy5* alleles in three different genetic backgrounds. Consistent with the earlier reports (Ang and Deng, 1994, Koornneef et al., 1980), all the *hy5* alleles showed significantly elongated hypocotyls at 22°C, both in long and short days (Figures S1D–S1G, S2A, and S2B). Whereas the hypocotyl response at 27°C was significantly reduced in short days (Figures S1D–S1G), it was comparable to wild-types in long days (Figures S2A and S2B). We have further confirmed that the temperature conditions in our experiments (22°C versus 27°C) are comparable with that of Delker et al. (2014) (20°C versus 28°C) (Figures S2C–S2E). DET1 is shown to regulate elongation growth by inhibiting HY5 function in the dark cooperatively with COP1 through protein degradation (Osterlund et al., 2000). Consistent with this, *cop1-4* and *cop1-6* alleles were defective in hypocotyl elongation, both at 22°C and 27°C similar to *det1-1* (Figures 1E and 1F). In addition, a transgenic line overexpressing *COP1* (*COP1*-OE) (Holm et al., 2001) showed significantly longer hypocotyl at 22°C, and enhanced temperature-responsive growth at 27°C (Figures 1E, 1F, S3A, and S3B), further confirming the positive role of COP1 in temperature-induced elongation growth.

Expression of auxin biosynthesis gene *YUC8* and genes involved in cell elongation such as *EXP8* and *XTR7* that underlie elongation growth in response to temperature showed robust upregulation in *Col-0* upon shift to 27°C (Figure 1G). The *det1-1* and *cop1-4* mutants showed strongly attenuated expression of these genes even at 27°C (Figure 1G), whereas *COP1-OE* showed elevated expression (Figures S3C–S3E). However, *hy5-215* showed increased expression of these genes both at 22°C and 27°C (Figure 1G), suggesting HY5 function is required to negatively regulate these genes. Transcription factor PIF4 has been shown to be the key activator of thermosensory growth. Expression of *PIF4* is robustly induced by elevated temperature, as shown before (Koini et al., 2009, Kumar et al., 2012). Interestingly, temperature-induced expression of *YUC8* correlated with that of *PIF4* in *det1-1* and *cop1-4* mutant, but not in *hy5-215* (Figure S1H). This was surprising given the recent finding that HY5 regulates elongation growth through transcriptional regulation of *PIF4*. To understand this further, we studied *PIF4* expression at regular intervals after exposure to 27°C. In line with the hypocotyl and gene expression phenotypes, *PIF4* expression in both *det1-1* and *cop1-4* was strongly attenuated and showed severely impaired temperature response (Figures 1I and S3F). Additionally, *PIF4* expression was strongly upregulated in *COP1*-OE (Figure S3F). These results clearly suggest that DET1 and COP1 are positive regulators of *PIF4* as reported earlier (Delker et al., 2014, Ma et al., 2016). These results indicate that transcriptional regulation of *PIF4* as a possible mechanism by which DET1/COP1 signaling control growth. Interestingly, in contrast to the earlier report (Delker et al., 2014), *PIF4* expression in the *hy5* mutant was comparable to that of Col-0 both at 22°C and 27°C at all time points (Figures 1I and S3G), suggesting that transcriptional regulation of *PIF4* is unlikely to be the mechanism by which HY5 regulates temperature-responsive elongation growth. The above results show that DET1 and COP1 are essential for hypocotyl growth and temperature-responsive elongation as reported earlier (Delker et al., 2014). However, contrary to what has been proposed, our data showed that DET1 and COP1, not HY5, function at least in part through regulating *PIF4* expression (Figures 1I and S3G). Together, these results suggested that DET1/COP1 and HY5 modulate PIF4-mediated growth possibly through distinct mechanisms.

### HY5 Is Dispensable for the Control of Thermosensory Elongation Growth by DET1 and COP1

**Figure 2:**
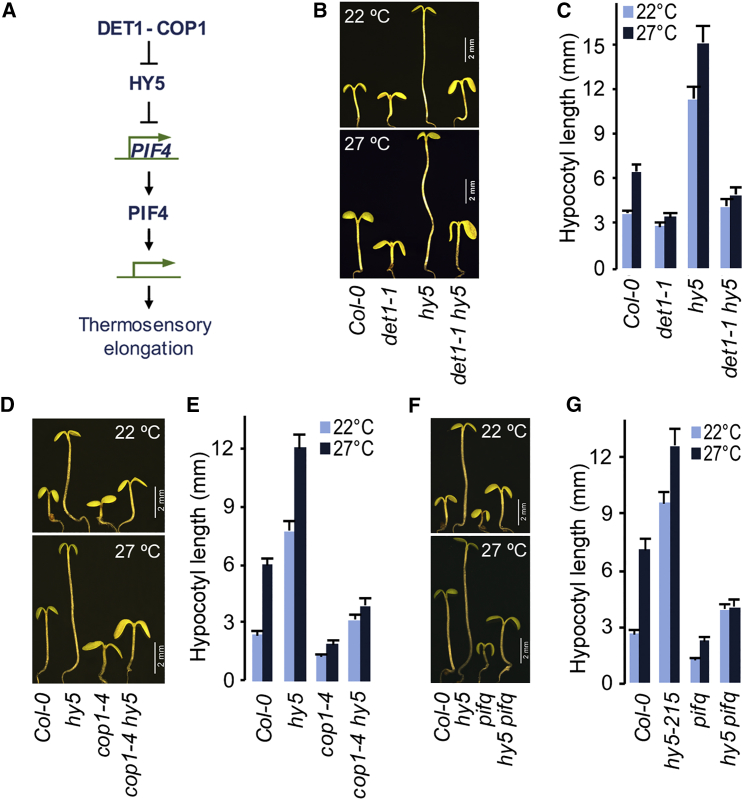
HY5 Is Not Essential for the Control of Thermosensory Elongation Growth by DET1 and COP1 (A) Linear hierarchical model showing the regulation of *PIF4* expression and PIF4-mediated thermosensory elongation growth by DET1/COP1-HY5 as proposed by Delker et al. (2014). (B and C) Control of thermosensory hypocotyl elongation by DET1 is not completely HY5 dependent showing that DET1 and HY5 control hypocotyl elongation through distinct mechanisms. Seedling hypocotyl picture (B) and hypocotyl length data (C) of 7-day-old seedlings grown in SD are shown (mean ± SD; n ≥ 20). (D and E) COP1-mediated thermosensory hypocotyl elongation is completely independent of HY5, suggesting that they control hypocotyl elongation through distinct mechanisms. Seedling hypocotyl picture (D) and hypocotyl length data (E) of 7-day-old seedlings grown in SD are shown (mean ± SD; n ≥ 20). (F and G) Loss of major PIFs strongly suppress *hy5* hypocotyl phenotype similar to that of loss of DET1 or COP1, suggesting that DET1 and COP1 regulates PIF-mediated elongation independently of HY5. Representative hypocotyl picture (F) and hypocotyl length (G) of seedlings grown in 22°C and 27°C SD for 7 days are shown. Data shown are mean ± SD (n ≥ 20).

The results above suggested that DET1/COP1 and HY5 may regulate thermosensory elongation growth through distinct mechanisms. In the regulation of seedling etiolation in the dark and de-etiolation in light, it has been shown that DET1 and COP1 exert their effect through regulating HY5 protein levels. It has been suggested to be the paradigm for regulation in temperature-induced hypocotyl elongation as well. A linear hierarchical pathway has been proposed where HY5, under regulation by DET1/COP1, transcriptionally regulate *PIF4* in order to regulate elongation growth (Delker et al., 2014) (Figure 2A). In light of our above results, we sought to examine the role of HY5 in DET1/COP1-mediated elongation growth at high ambient temperature. We hypothesized that if HY5 is required for DET1/COP1-mdiated hypocotyl growth, loss of HY5 function in *det1-1* and *cop1-4* mutants should significantly, if not completely, suppress the short hypocotyl and the lack of elongation response at elevated temperature. Conversely, if DET1 and COP1 function independently of HY5, their loss of function should significantly suppress elongation phenotypes of *hy5* mutant. We therefore assessed hypocotyl elongation of *det1-1 hy5* and *cop1-4 hy5* double mutants. Consistent with the above results and our hypothesis of distinct regulatory roles, both *det1-1* and *cop1-4* strongly suppressed *hy5* phenotype in double mutants. In addition, both *det1-1 hy5* and *cop1-4 hy5* double mutants showed severely attenuated temperature-responsive hypocotyl elongation (Figures 2B–2E). These findings are consistent with the earlier studies suggesting that HY5 alone is not sufficient to explain the regulation of photomorphogenesis by DET1 and COP1(Ang and Deng, 1994, Chory, 1992, Fernando and Schroeder, 2015). Similarly, the *hy5 pifq* quintuple mutant (Jia et al., 2014) strongly phenocopied *det1 hy5* and *cop1 hy5* (Figures 2F and 2G), suggesting that DET1 and COP1 primarily act to maintain PIF function. Together, these results further confirm our hypothesis that DET1/COP1 and HY5 regulate temperature-induced elongation growth through independent mechanisms.

### DET1 and COP1 Positively Regulate Thermosensory Elongation through Promoting PIF4 Protein Abundance

**Figure 3:**
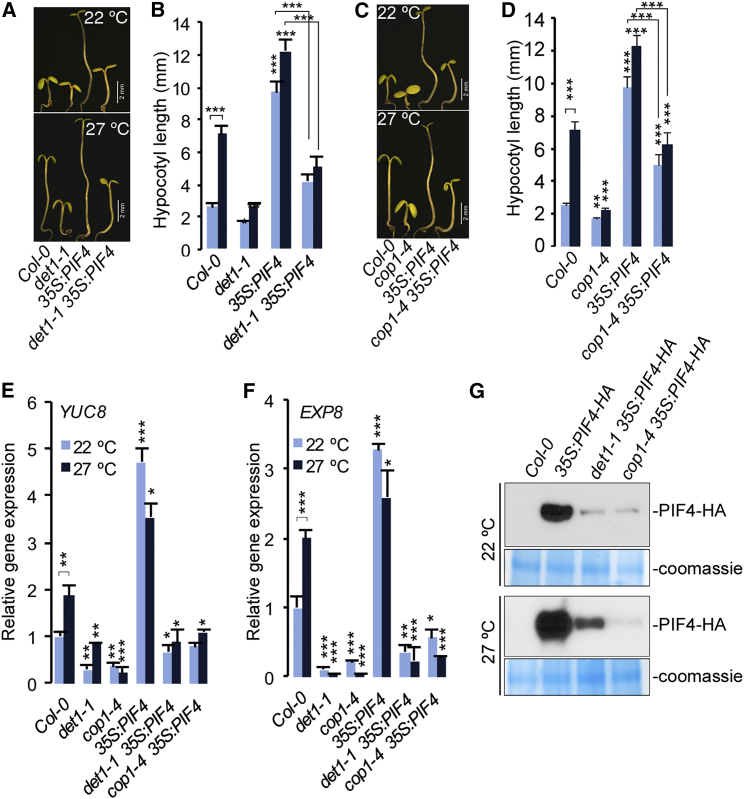
DET1 and COP1 Positively Regulates Elongation through Promoting PIF4 Abundance (A) *det1-1* suppresses hypocotyl elongation phenotype of *35S:PIF4*. Representative seedling pictures of 7-day-old Col-0, *det1-1*, *35S:PIF4*, *and det1-1 35S:PIF4* grown at constant 22°C and 27°C under short-day photoperiod are shown. (B) Seedling hypocotyl measurement data (mean ± SD; n ≥ 20) of genotypes shown in (A). (C) *cop1-4* strongly suppresses elongated hypocotyl phenotype of *35S:PIF4*. Seven-day-old representative seedling hypocotyl pictures of Col-0, *cop1-4*, *35S:PIF4*, and *cop1-4 35S:PIF4* grown at constant 22°C and 27°C under short-day photoperiod. (D) Seedling hypocotyl measurement data (mean ± SD; n ≥ 20) of genotypes shown in (C). (E and F) Expression of PIF4 target genes *YUC8* (E) and *EXP8* (F) in Col-0, *det1-1*, *cop1-4*, *35S:PIF4*, *det1-1 35S:PIF4* and *cop1-4 35S:PIF4* as measured by qRT-PCR (mean ± SD of three biological replicates) in 7-day-old seedlings grown at 22°C and after 24-hr incubation at 27°C. (G) Immunoblot showing the abundance of PIF4-HA protein in 7-day-old Col-0 (used as negative control), *35S:PIF4-HA*, *det1-1 35S:PIF4-HA*, and *cop1-4 35S:PIF4* seedlings grown in 22°C short days or treated with 27°C for 24 hr. See also Figure S3. In (B), (D), (E), and (F), ^∗^p ≤ 0.05, ^∗∗^p ≤ 0.01, ^∗∗∗^p ≤ 0.001 (Student’s t test) significantly different from Col-0 in corresponding temperature conditions or between indicated pairs.

We found that while DET1 and COP1 were essential for PIF4-mediated elongation growth, *PIF4* expression was attenuated in *det1-1* and *cop1-4* mutants, and was upregulated by *COP1* overexpression suggesting that DET1 and COP1 exert their effect at least in part through positively regulating *PIF4* expression. To test whether transcriptional regulation of *PIF4* by DET1 was sufficient to control thermosensory elongation growth, we introduced *35S*:*PIF4-HA* (Nozue et al., 2007) into *det1-1*. *35S*:*PIF4* leads to exaggerated hypocotyl elongation (de Lucas et al., 2008) and should suppress the elongation phenotype of *det1-1*, if DET1 functions mainly to control *PIF4* expression. Interestingly, *det1-1* mutation strongly suppressed the long hypocotyl phenotype of *35S:PIF4* and severely attenuated temperature-responsive growth at 27°C (Figures 3A and 3B). Similarly, we found that *cop1-4* also strongly suppressed the long hypocotyl phenotype of *35S:PIF4* (Figures 3C and 3D). Further, expression of elongation-related genes *YUC8* and *EXP8* was strongly suppressed in *det1-1 35S*:*PIF4-HA* and *cop1-4 35S:PIF-HA* to levels comparable to *det1-1* and *cop1-4*, respectively (Figures 3E and 3F), despite strong *PIF4* overexpression (Figure S3H), suggesting that DET1 and COP1 also regulate PIF4 post-transcriptionally.

PIF4 function is controlled by environmental signals through modulation at the protein level (de Lucas et al., 2008, Leivar and Monte, 2014, Xu et al., 2015). Consistent with earlier studies (Dong et al., 2014), we found that PIF4-HA protein abundance was severely reduced in *det1-1* and *cop1-4* backgrounds (Figure 2G). These results further confirm that DET1 and COP1 control PIF4-mediated elongation growth through stabilizing PIF4 protein. The exact mechanism through which DET1 stabilizes PIF4 protein is not known at this point; this could be possibly through modulating photoreceptor or DELLA protein functions (Li et al., 2015). These results provide further support to our hypothesis that DET1 functions independently of HY5 to regulate elongation growth.

### HY5 Negatively Regulates PIF4-Mediated Thermosensory Gene Induction

**Figure 4:**
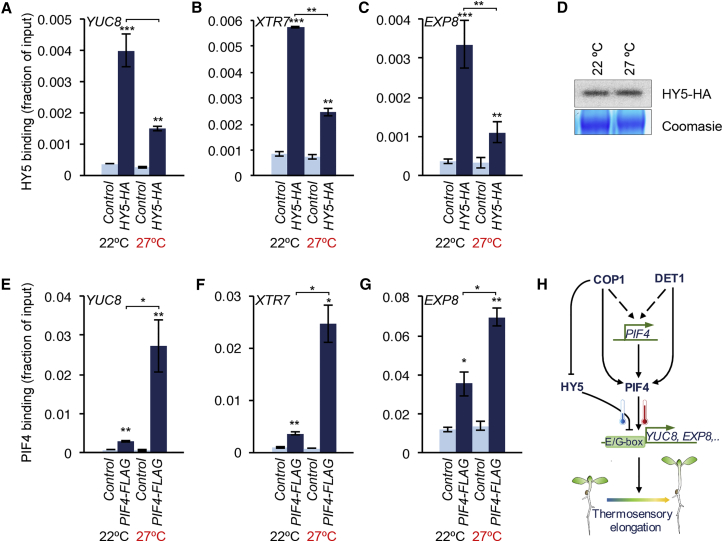
Integration of Regulatory Mechanisms for PIF4-Mediated Thermosensory Growth (A–C) HY5 directly binds to the promoters of PIF4 targets involved in elongation growth. Chromatin immunoprecipitation of HY5 using *35S:HY5-HA* seedlings (either grown at constant 22°C or treated with 27° for 24 hr) and wild-type (used as negative control) shows enrichment at *YUC8* (A), *XTR7* (B), and *EXP8* (C) promoters (mean ± SD, n = 3). ^∗^p ≤ 0.05, ^∗∗^p ≤ 0.01, ^∗∗∗^p ≤ 0.001 (Student’s t test) significantly different from wild-type (WT) in corresponding temperature conditions or between indicated pairs. See also Figure S4. (D) Accumulation of HY5-HA protein as shown by immunoblot in 7-day-old *35S:HY5-HA* seedlings grown in 22°C short days or treated with 27°C for 24 hr. (E–G) Binding of PIF4 to genes responsible for elongation growth. Chromatin immunoprecipitation of *35S:PIF4-HA* (as a positive control) and wild-type (used as negative control) showing enrichment at *YUC8* (E), *XTR7* (F), and *EXP8* (G) promoters (mean ± SD, n = 3). ^∗^p ≤ 0.05, ^∗∗^p ≤ 0.01, ^∗∗∗^p ≤ 0.001 (Student’s t test) significantly different from WT in corresponding temperature conditions or between indicated pairs. See also Figure S4. (H) Mechanism of DET1- and COP1-mediated regulation of thermosensory growth. Both DET1 and COP1 are found to be essential to stabilize PIF4 and thereby promote thermosensory elongation growth. PIF4 bind to the promoters of growth genes such as *YUC8*, *EXP8*, etc. and activate their expression leading to the elongation growth. HY5 on the other hand negatively regulates thermosensory growth by competing with PIF4 for binding to the promoters of growth genes and suppressing their expression. Elevated temperature leads to a decrease in HY5 activity resulting in reduced HY5 binding thus results in activation of PIF4 target genes.

Together, the above results confirm that DET1 and COP1 modulate thermosensory growth by promoting PIF4 function directly. Despite its strong influence on hypocotyl elongation and expression of PIF4 target genes, we found that HY5 did not seem to regulate *PIF4* expression (Figures 1I and S3G). We therefore hypothesized that HY5 could directly modulate activation of target genes by PIF4. It has been previously shown that HY5 binds to conserved E/G box elements and competes with PIF transcription factors at ROS-responsive (Chen et al., 2013) and photo-pigment biosynthetic genes (Toledo-Ortiz et al., 2014). To test whether HY5 targets elongation genes underlying thermosensory growth, we performed chromatin immunoprecipitation of HY5-HA protein in seedlings grown at 22°C or were shifted to 27°C for 24 hr. We found that HY5-HA binds to the same promoter regions of *YUC8*, *XTR7*, and *EXP8*, confirming that the genes controlling thermosensory elongation response are directly regulated by HY5. Moreover, at elevated temperature (27°C) HY5 binding to all the tested elongation-related gene promoters was significantly decreased (Figures 4A–4C and S4). Immunoblot analysis has shown that this temperature-dependent chromatin binding is not due to altered HY5 abundance (Figure 4D). It has been well established that PIF4 binds directly to the chromatin of *YUC8*, *XTR7*, and *EXP8* promoters as reported (Nieto et al., 2015, Sun et al., 2012) and that PIF4 binding to chromatin is temperature dependent. Using a P_*PIF4*_:*PIF4-FLAG* transgenic line in our chromatin immunoprecipitation sequencing (ChIP) experiments, we show that PIF4 binds to the promoters of *YUC8*, *XTR7*, and *EXP8*, and most importantly this binding was temperature responsive with increased binding observed at 27°C (Figures 4E–4G and S4). These results show that HY5 could potentially compete with PIF4 for binding the promoters of elongation-related genes at lower temperatures. PIF4 binds and activates its targets in a temperature-dependent manner, with increased binding at elevated temperature (Franklin et al., 2011, Kumar et al., 2012). Modulation of HY5 function can therefore fine-tune PIF4-mediated elongation growth in a temperature-dependent manner. This potentially antagonistic regulation of growth genes by HY5 and PIF4 provides a possible molecular mechanism for coordinated gene regulation in response to environmental signals.

In summary, our data demonstrate that DET1/COP1 and HY5 control PIF4-mediated elongation growth through independent mechanisms (Figure 4H). DET1 and COP1 promote PIF4 function primarily through stabilizing the protein, in addition to maintaining *PIF4* expression. Thus, DET1/COP1 act as the core positive regulatory module for PIF4 function regardless of temperature. Temperature-dependent modulation of DET1 and COP1 function could therefore directly control thermosensory growth independent of HY5. Indeed, inhibition of HY5 function by DET1/COP1 adds an additional layer of regulation. Importantly, we find that HY5 controls PIF4-mediated elongation mainly through competitive binding at the promoter chromatin and repression of PIF4 target genes, but not through transcriptional repression of *PIF4*. This competitive inhibition is significantly reduced with increased temperature. At elevated temperature HY5 protein becomes less abundant (Toledo-Ortiz et al., 2014), likely through increased COP1 activity leading to removal of repression, coinciding with elevated PIF4 accumulation and target gene activation. The regulatory model we propose is also supported by long established genetic evidence that HY5 alone could not explain regulation of elongation growth by DET1 and COP1 (Ang and Deng, 1994, Chory, 1992). Collectively, this study provides a mechanistic framework where multiple regulatory modules cooperatively control growth through integrating environmental inputs such as light and temperature. Elucidating the coordinated control of growth in an ecological context is important for understanding phenotypic plasticity and adaptation, especially in the context of global climate change that threatens biodiversity in the wild and productivity in the field alike (Battisti and Naylor, 2009).

## Experimental Procedures

### Plant Materials and Growth Conditions

Unless otherwise specified, Columbia ecotype (Col-0) was used in all the experiments. The *hy5* mutants, *hy5-215* (Datta et al., 2007) and *hy5* (Jia et al., 2014) are in (Col-0), whereas *hy5 ks-50* (Holm et al., 2002) and *hy5-1* (Datta et al., 2007) are in Wassilewskija (Ws) and Landsberg erecta (Ler-0) ecotypes, respectively. The *det1-1*, *cop1-4*, *cop1-6*, *hy5 cop1-4*, *hy5 det1*, and *pifq hy5* mutants have been described previously (Fernando and Schroeder, 2015, Holm et al., 2002, Jia et al., 2014, Schroeder et al., 2002). *det1-1* and *cop1-4* were crossed to *35S:PIF4-HA* (Nozue et al., 2007) to generate *det1-1 35S:PIF4-HA* and *cop1-4 35S:PIF4-HA* lines.

Seeds were surface-sterilized (70% ethanol + 0.5% Triton X-100) and germinated on Murashige and Skoog (MS) plates containing 0.5% sucrose following stratification for 3 days at 4°C. Upon germination at 22°C, seedlings were either transferred to 27°C or retained at 22°C for 7 days unless otherwise specified. The light intensity of 150 μmol m^–2^ s^–1^ was used to grow seedlings for all the experiments. Experiments were performed under short-day (8-hr-light/16-hr-dark) or long-day (16-hr-light/8-hr-dark) conditions as specified.

### Hypocotyl Measurement

Seven-day-old seedlings grown as specified above were used for hypocotyl measurements. At least 20 seedlings were imaged and hypocotyl lengths were measured using NIH ImageJ software (https://imagej.nih.gov/ij/).

### RNA Isolation and Gene Expression Analysis

RNA was extracted using RNeasy Plant mini kit (QIAGEN) (following manufacturer’s instructions). Approximately 1.5 μg of total RNA was converted into cDNA using Superscript III reverse transcriptase (Invitrogen). cDNA was diluted 1:25 and 2.0 μL was used for qPCR on Lightcycler 480 using SYBR Green Master Mix (Roche). Ef1α (AT5G60390) was used as control for normalization. Oligonucleotide sequences used are provided in Table S1.

### Immunoblot Analysis

For immunoblot analysis, seedlings were grown at 22°C for 7 days and were either transferred to 27°C for 24 hr or retained at 22°C. Tissue were harvested in dim light at dawn in all cases. Horseradish peroxidase (HRP)-conjugated hemagglutinin (HA) antibody (Miltenyi Biotech) was used to detect PIF4-HA and HY5-HA and visualized by chemiluminscent detection using Immobilon Chemiluminescent HRP substrate (Millipore).

### Chromatin Immunoprecipitation Analysis

ChIP was carried out as described (Kumar et al., 2012) with minor modifications. *P*_*PIF4*_*:PIF4-FLAG* and *35S:HY5-HA* seedlings and the respective controls (Col-0 and Ws, respectively) were grown on one-half MS plates for 6 days; they were either retained at 22°C or transferred to 27°C for 24 hr. Seedlings (∼2.0 g) were harvested in dim light and directly cross-linked with 1% formaldehyde. ChIP was done using paramagnetic μMACS beads coated with monoclonal anti-FLAG or anti-HA antibody (Milteney Biotech) according to manufacturer’s instructions. Beads were washed four times with the immunoprecipitation buffer followed by two washes with Tris-EDTA buffer (TE). Reverse cross-linking was done by boiling at 95°C for 10 min in presence of 10% Chelex (Bio-Rad) followed by proteinase K treatment at 50°C. qPCR was performed using Roche Light cycler and enrichment was calculated relative to wild-type controls. PIF4-HA and HY5-HA binding to *YUC8*, *EXP8*, and *XTR7* were performed using a set of primers spanning the promoter regions covering either E/G-box elements. Oligonucleotide sequence details are provided in Table S1.

### Statistical Analysis

For measuring hypocotyl length, unless otherwise mentioned at least 20 seedlings were used. For gene expression analysis, three independent biological replicates in combination with three technical replicates were used. All experiments were repeated at least thrice. The statistical significance between or among treatments and/or genotypes was determined based on Student’s t test.

## Author Contributions

S.N.G. designed and performed most of the experiments and analyzed data. S.V.K. designed and supervised the study, performed experiments, and analyzed data. S.N.G. and S.V.K. wrote the paper.

## Supplemental Information

Document S1. Figures S1–S4 and Table S1

Document S2. Article plus Supplemental Information
