# Supplementary material for: DET1 and HY5 Control PIF4-Mediated Thermosensory Elongation Growth through Distinct Mechanisms
Source: Cell Rep. 2017 Jan 10;18(2):344–51. doi: 10.1016/j.celrep.2016.12.046 (PMC5263232; doi:10.1016/j.celrep.2016.12.046)
Supplement: Document S1. Figures S1–S4 and Table S1 [file mmc1.pdf]

Cell Reports, Volume *18*

Supplemental Information

DET1 and HY5 Control PIF4-Mediated Thermosensory Elongation Growth through Distinct Mechanisms

Sreeramaiah N. Gangappa and S. Vinod Kumar

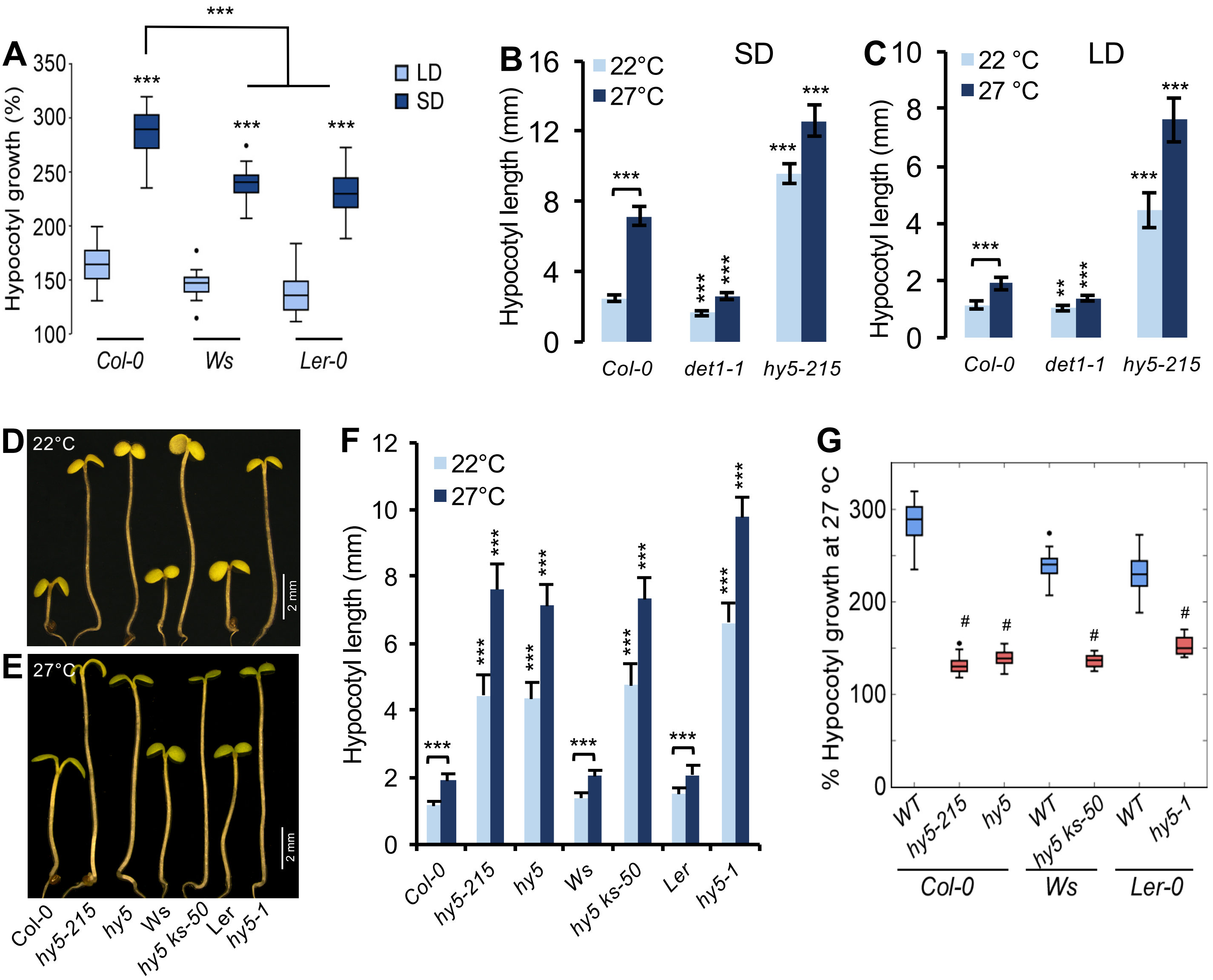

Figure S1. Thermosensory hypocotyl growth is photoperiod dependent. Related to Figure 1.
(**A**) Percent hypocotyl growth response (27 ºC to 22 ºC) of Col-0, Ws, and Ler-0 ecotypes grown under long-days (LD) and short-days (SD) for seven-days. Data shown is mean±SD (n≥20). ***P≤0.001 (Student's *t*-test) significantly different from LD in corresponding ecotypes or between indicated pairs as shown. (**B** and **C**) Hypocotyl length measurement data (mean±SD; n≥20) of Col-0, *det1-1,* and *hy5-215* mutants grown in 22 ºC and 27 ºC SD (B) and LD (C) for seven-days. (**D-G**) Representative seedling pictures of various *hy5* mutant alleles and their corresponding Wild-Types grown at constant 22 ºC (D) and 27 ºC (E) under short-days for seven-days. (**F** and **G**) Hypocotyl elongation measurement data (F) (mean±SD; n≥20) and % hypocotyl growth (27 ºC to 22 ºC) (G) of genotypes shown in (D) and (E). #P≤0.0001 (Student's *t*-test) significantly different from corresponding wild-type strains.
In Figure S1B, S1C, and S1F, **P≤0.01, ***P≤0.001 (Student's *t*-test) significantly different from Col-0 in corresponding temperature conditions or between indicated pairs.

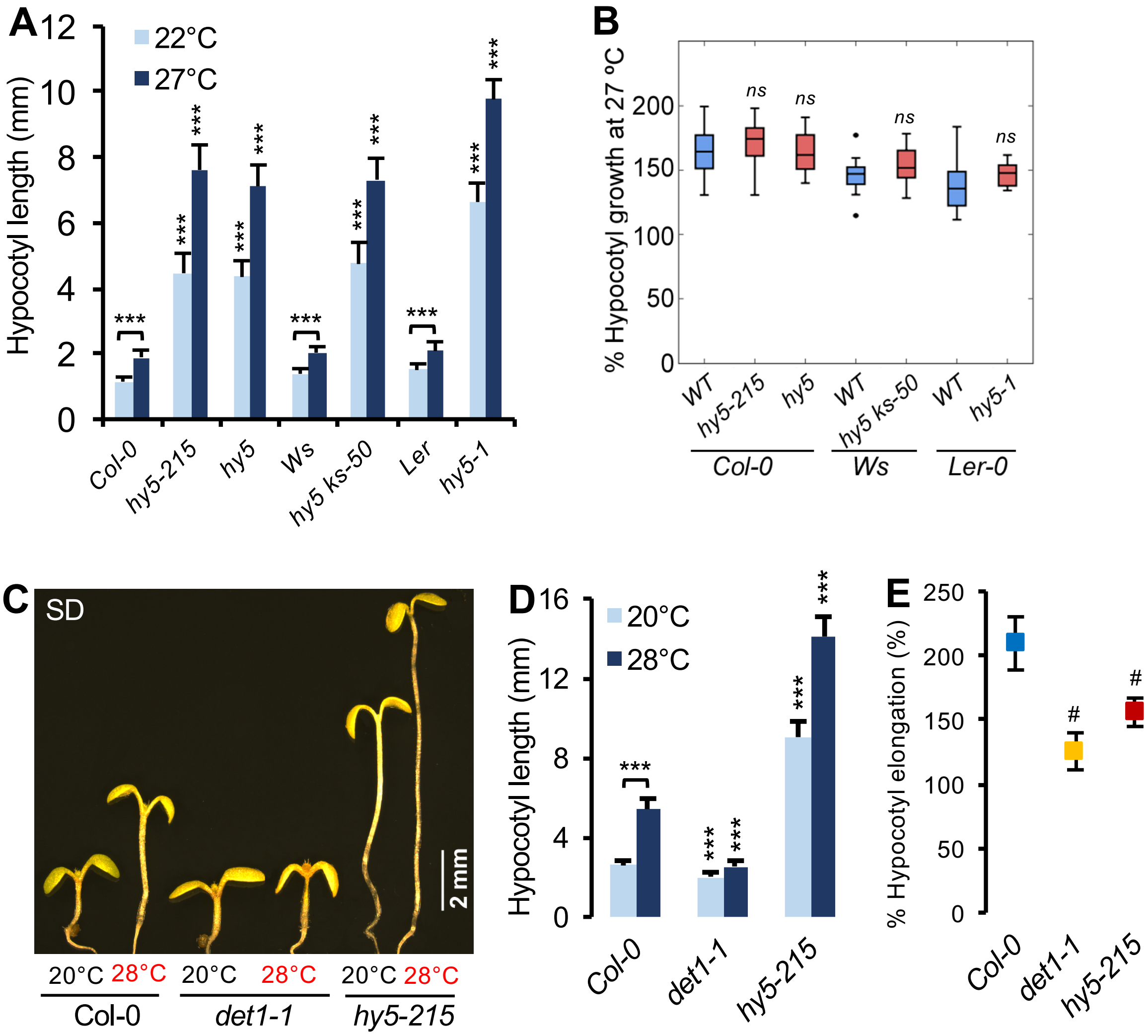

Figure S2. Control of thermosensory hypocotyl growth by DET1 and HY5. Related to Figure 1.

(**A** and **B**) Hypocotyl measurement data (A) (mean±SD; n≥20) and percent hypocotyl growth (B) of various *hy5* alleles grown at constant 22 and 27 ºC under LD for seven-days. ***P≤0.001 (Student's *t*-test) significantly different from corresponding wild-types in corresponding temperature conditions or between indicated pairs. *ns*, not-significantly different from the respective wild-types. (**C-D**) Representative seedling hypocotyl picture (C), hypocotyl elongation measurement data (D) (mean±SD; n≥20). ***P≤0.001 (Student's *t*-test) significantly different from Col-0 in corresponding temperature conditions or between indicated pairs. (**E**) Relative hypocotyl response (%) of the genotypes shown in (C). #P≤0.001 (Student's *t*-test) significantly different from corresponding wild-type strains.

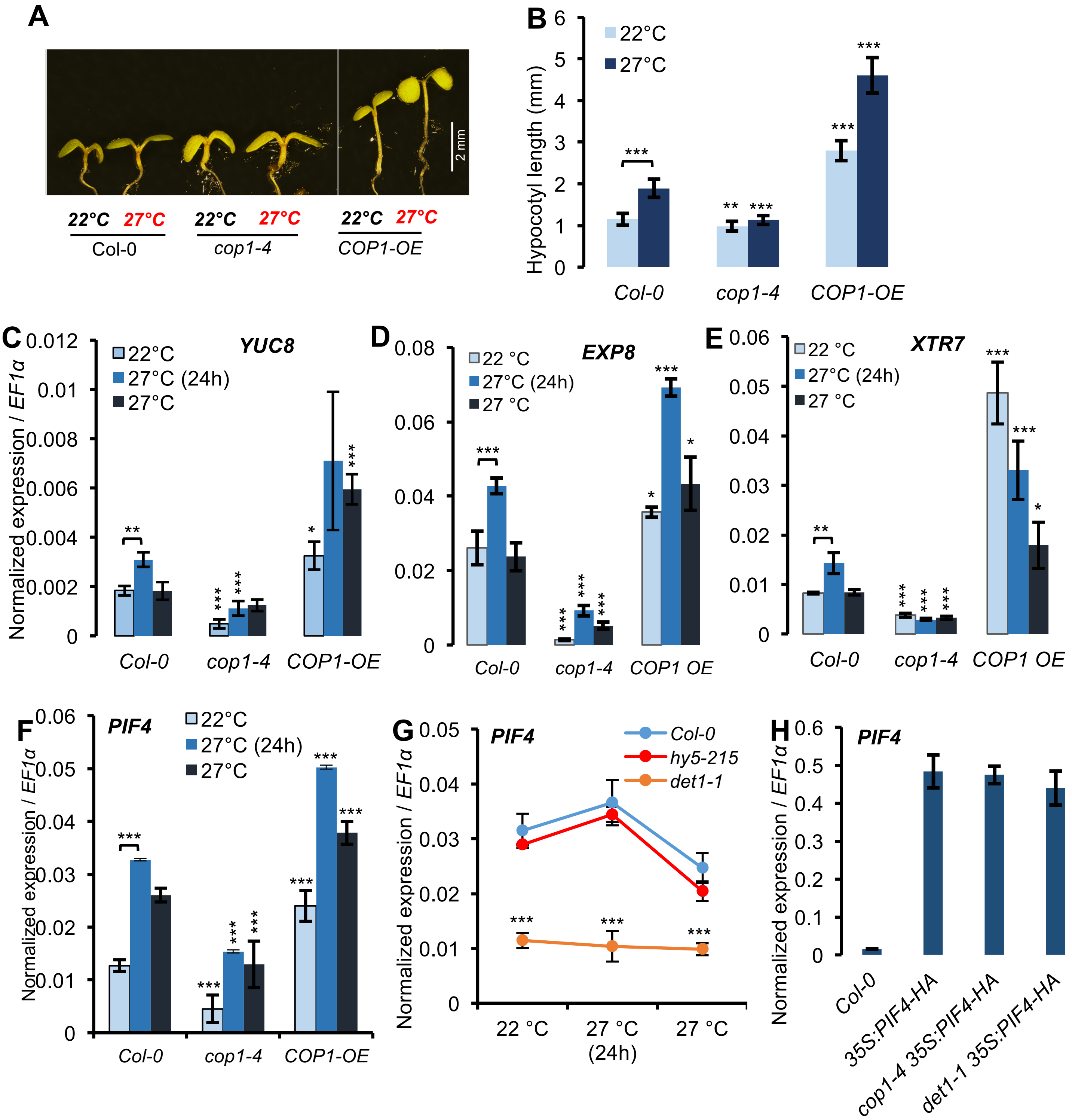

Figure S3. Thermosensory hypocotyl growth and the expression of elongation-related genes. Related to Figure 1 and Figure 3.

(**A** and **B**) Representative seedling hypocotyl picture (A) and measurement data (B) (mean±SD; n≥20) of Col-0, *cop1-4*, and *COP1-OE* lines in 22 ºC and 27 ºC for seven-days in LD. (**C-F**) Expression of growth responsive genes *YUC8* (C), *EXP8* (D), and *XTR7* (E); and *PIF4* (F) are downregulated in *cop1-4* mutant, but upregulated in *COP1-OE* compared to Col-0 as measured by qRT-PCR (mean±SD of three biological replicates) in seven-day-old seedlings grown constantly at 22 ºC, after 24 h incubation at 27 ºC, as well as continuous growth at 27°C in short day conditions. (**G**) Expression of *PIF4* in Col-0, *hy5-215*, and *det1-1* as measured by qRT-PCR (mean±SD of three biological replicates) in seven-day-old seedlings grown at constant 22 ºC and 27 ºC, and 27 ºC adapted seedlings for 24h in LD conditions. (**H**) Expression of *PIF4* in *35S:PIF4-HA*, *cop1-4 35S:PIF4-HA,* and *det1-1 35S:PIF4-HA* as measured by qRT-PCR (mean±SD of three biological replicates) in seven-day-old seedlings grown at constant 22 ºC SD conditions. In Figure S3B-S3G, *P≤0.05, **P≤0.01, ***P≤0.001 (Student's *t*-test) significantly different from Col-0 in corresponding temperature conditions or between indicated pairs.

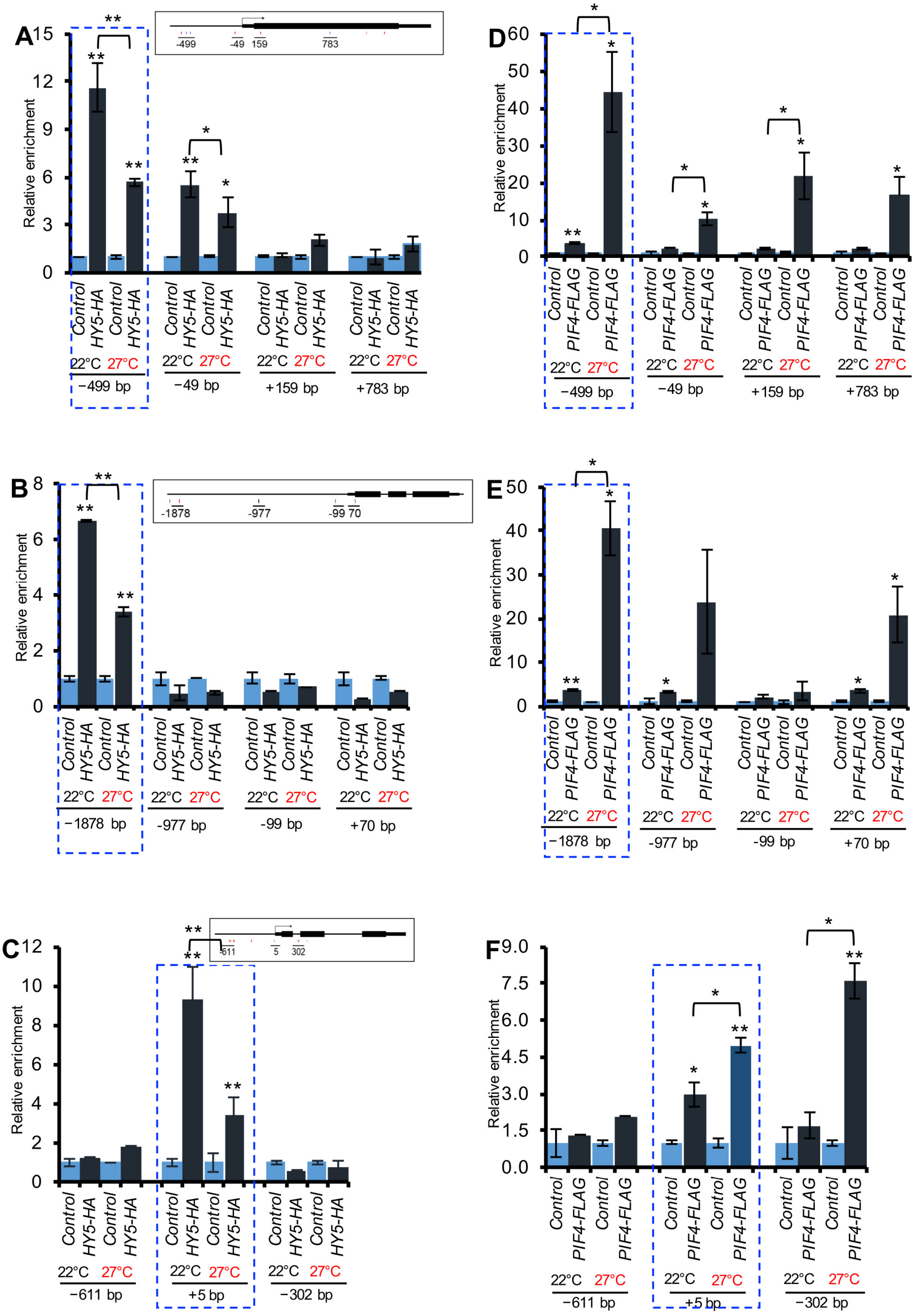

Figure S4. HY5 binds to the PIF4 targets. Related to Figure 4.

(A-F) ChIP analysis showing binding of HY5 (A-C) and PIF4 (D-F) to the PIF4 targets such as *YUC8* (A and D), *XTR7* (B and E), *EXP8* (C and F) as measured by by qRT-PCR (mean±SD of two biological replicates) in seven-day-old seedlings grown at constant 22 ºC, and 27 ºC adapted seedlings for 24 h in short day conditions. Insets in A, B and C show gene diagram along with position of *G*-box (blue line) and/or *E*-box (red line) elements present in the promoters, and position of primer pairs used for Q-PCR. *P≤0.05, **P≤0.01, ***P≤0.001 (Student's *t*-test) significantly different from wild-type (Control) in corresponding temperature conditions or between indicated pairs. Marked area with the blue dotted line indicate the regions that are shown in the Figure 4A-4C and 4E-4G, respectively for *HY5-HA* and *PIF4-FLAG*.

Table S1. Oligo nucleotide sequences used in the study. Related to Figure1, Figure3 and Figure4

**Name**	**Oligo No.**	**Oligo Sequence (5'………….3')**	**Purpose**
**Primers used for gene expression analysis**			
EXP8-F	633	CTCTTTCCGAAGAGTACCATGT	Q-PCR
EXP8-R	634	GTGTACGTCTCCTGCTCCTC	Q-PCR
YUC8-F	615	CGATGAGACCAGTGGCTTGT	Q-PCR
YUC8-R	616	TTTTCTCCCGTAGCCACCAC	Q-PCR
PIF4-F	766	ACCTCAGAGACGGTTAAGCC	Q-PCR
PIF4-R	767	TGGAGGAGGCATGACTTGAG	Q-PCR
ATHB2-F	667	CCGTCGGCTACAAAAAGAAG	Q-PCR
ATHB2-R	668	GAAGGGCACATGGTCAAAGT	Q-PCR
XTR7-F	1127	CGGCTTGCACAGCCTCTT	Q-PCR
XTR7-R	1128	TCGGTTGCCACTTGCAATT	Q-PCR
**Primers used for ChIP analysis**			
YUC8-F (-499 bp)	1294	GGGAATGGGTTTGATGTGGA	Q-PCR
YUC8-R (-499 bp)	1295	GGGTGATTCTTTGTGGGACC	Q-PCR
YUC8-F (-49 bp)	1296	TCGTCGAGTGGAAAAATATTCA	Q-PCR
YUC8-R (-49 bp)	1297	TGGAAATGGTTTTGAATTTGG	Q-PCR
YUC8-F (+159 bp)	1298	GGAGAATATGTTTCGTTTGATGG	Q-PCR
YUC8-R (+159 bp)	1299	CTAACCCCGACGGTCCAG	Q-PCR
YUC8-F (+783 bp)	1300	AGTTTCTCTTGACCTAGCAAACC	Q-PCR
YUC8-R (+783 bp)	1301	AACATCTTCATTGCAAGCTCAA	Q-PCR
EXP8-F (-611 bp)	1286	AAACCTTCAACAAAAATGTGAGG	Q-PCR
EXP8-R (-611 bp)	1287	ACATGCAAATGTTGCGGTTA	Q-PCR
EXP8-F (-611 bp)	1288	GGCACAATTATTCACTCGAGTAC	Q-PCR
EXP8-R (-611 bp)	1289	AGGAGGGGTTAGTTATTTCGGT	Q-PCR
EXP8-F (-611 bp)	1290	GGGTACGTATGACCAATCCCA	Q-PCR
EXP8-R (-611 bp)	1291	GTGTTCGTCCCGTAACCTTG	Q-PCR
XTR7-R (-1878 bp)	1336	GGATTTCATTGTATTTGCATGCC	Q-PCR
XTR7-R (-1878 bp)	1337	TAGTGCTACATCCGACGTGT	Q-PCR
XTR7-R (-977 bp)	1338	TGGTTTCAATAGACGATCCTTGT	Q-PCR
XTR7-R (-977 bp)	1339	CGTCGACCACACCATTCTCT	Q-PCR
XTR7-R (-99 bp)	1340	GGAATTTTAGAGATTTTCTCTCATCTG	Q-PCR
XTR7-R (-99 bp)	1341	GCATTGGGAAGCTAAGACCA	Q-PCR
XTR7-R (+70 bp)	1342	ACACCAAAACACAAAGCTCTCA	Q-PCR
XTR7-R (+70bp)	1343	CAAGAAGAACAGTCGCCACG	Q-PCR
